# Combined α-thalassemia and Hemoglobin J-Iran (β77 His → Asp). A Family Study in southern Iran

**Published:** 2011-08-01

**Authors:** S J Dehghani, A Amiri Dashtarzhen, Sh Nasirabadi, J Dehbozorgian, A R Afrasiabi, N Morshedi, J Imanifard, Sh Mehrpoor, J Gerdabi, M Karimi

**Affiliations:** 1 Hematology Research Center, Shiraz University of Medical Science, Shiraz, Iran

**Keywords:** Hb J-Iran, α-thalassemia, Hemoglobin variants, Electrophoresis, High performance liquid chromatography

## Abstract

We report a 23-year-old man and three members of his family with Hb J-Iran confirmed by electrophoresis, chain separation by high performance liquid chromatography and sequencing. Alpha thalassemia was also confirmed in two family members. The substitution at β77 led to a higher negative charge of the βJ-Iran subunit, which enhanced its electrostatic attraction for the normal positively-charged α subunit. Therefore, more Hb J-Iran than Hb A forms in the red blood cells of heterozygotes. In α-thalassemia, the more attractive βJ-Iran subunit outcompetes βA subunits in forming assemblies with deficient α subunits, so even more Hb J-Iran was formed.

## Introduction

Hb J-Iran (β77 His→Asp) is one of the hemoglobin variants that initially have been discovered in Iran. The substitution at β77 leads to a higher negative charge on the surface of the βJ-Iran subunit, which enhances its electrostatic attraction for the normal positively, charged α subunit. As a result, red cells in heterozygous individuals contain more Hb J-Iran than Hb A.

## Case report

**Fig. 1:**
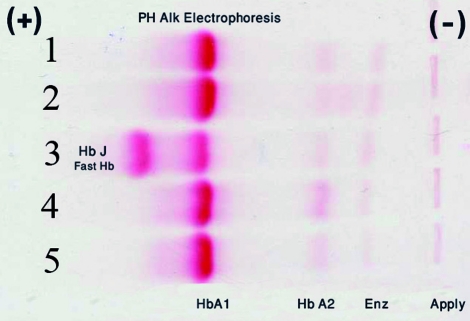
Hb electrophoresis of the proband (Patient no. 3) at pH: 8.6 detected a band located between Hb A1 and Hb H.

A 23-year-old man with mild jaundice and anemia was referred to the Hematology Research Center and Genetic and Prenatal Diagnosis of Thalassemia and Hemophilia Service of Datgheib and Nemazee Hospital (the main reference hospitals affiliated to Shiraz University of Medical Sciences in Shiraz, southern Iran) for premarital screening for thalassemia. On physical examination, he was asymptomatic and had no clinical manifestations. However, hemoglobin electrophoresis at pH 8.6 (Helena, Process-24, Beaumont, TX, USA) detected a band located between Hb A1 and Hb H (Figure 1). The proportion of this unknown band was determined by capillary electrophoresis (Sebia, Capillarys 2, and Norcross, GA, USA).

**Fig. 2:**
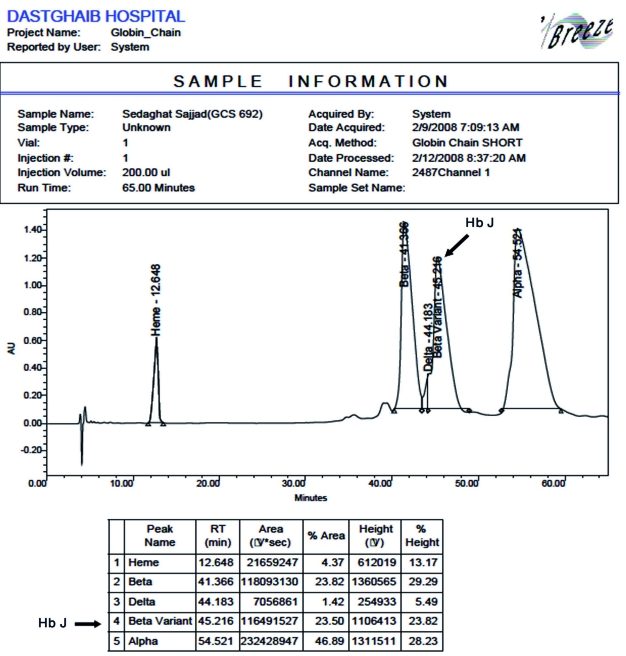
Chain separation in the proband by HPLC. The β chain retention time was shorter than the α chain time, and because the total proportion of the βA and variants was almost equal to the proportion of total α chain, we concluded that the variant was a β variant.

**Fig. 3:**
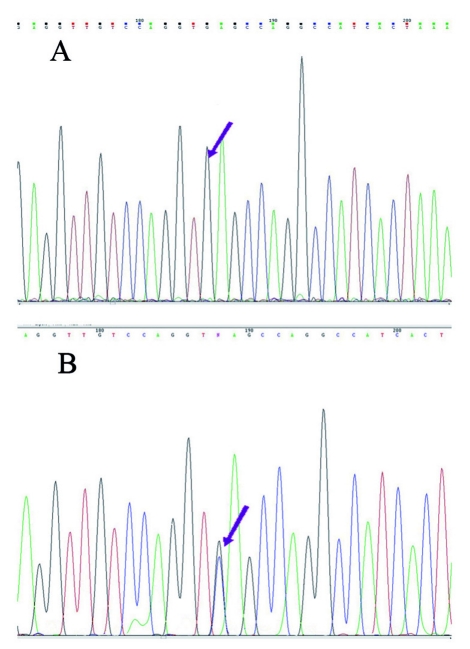
β gene sequence in the proband (B) compared to a normal sequence (A). A mutation was detected in codon 77 (CAC >GAC), which caused the β77 His→Asp substitution in the β chain (Hb J- Iran). The arrows show codon 77.

To determine the nature of the unknown Hb, chain separation by high performance liquid chromatography (HPLC) was used. The lysate was injected in a Vydac column in a Waters system (Waters, Breeze, Milford, MA, USA), and comparison of the retention times identified the unknown band as a β variant (Figure 2). The β gene was sequenced with an ABI 310 Genetic Analyzer (Applied Biosystems, Carlsbad, CA, USA) to detect the mutation or deletion which caused the formation of this hemoglobin. By comparing sequencing graphs for normal and sample sequences, we detected a mutation in codon 77 (CAC >GAC) which caused the β77 His→Asp substitution in the β chain (Hb J-Iran) (Figure 3). Molecular studies of the alpha globin gene found the homozygous form of the 3.7-kbp deletion (-α(-3.7)/-α(-3.7)), the probable cause of mild hypochromic microcytic anemia in the proband (diagnosed by blood smear prepared from fresh blood and stained with Wright stain). To detect long deletions in the alpha gene, gap- PCR was done, and to detect mutations or deletions, the reverse dot blot method was used according to the manufacturer’s protocol for the strip assay α globin gene kit (Viennalab, Vienna, Austria). Further studies were done in five of the proband’s siblings and his mother (his father had died before he was referred to our center). The results are summarized in Table 1.

**Table 1:** Hematological results in the proband and family members a

	**Mild hemochromic microcytic anemia**	**Hb (g/dL)**	**Hct (%)**	**MCV (fL)**	**MCH (pg)**	**MCHC (g/dL)**	**RBC count (10^6^)**	**Presence of J-Iran Hb**	**Amount of J-Iran Hb**	**Genotype of α-globin chain **
Proband	Yes	13.2	43.0	72.0	22.1	30.7	5.97	Yes	52.2	-α^-3.7^/-α^-3.7^
Mother	No	12.2	41.8	87.1	25.4	29.2	4.80	Yes	54.3	-α^-3.7^/αα
1st sister	No	13.4	44.4	86.2	26.0	30.2	5.15	Yes	49.8	αα/αα
2nd sister	No	13.8	44.9	90.7	27.9	30.7	4.95	Yes	49.5	αα/αα
3rd sister	No	13.4	43.3	86.9	26.9	30.9	4.98	No	ND	αα/αα
1st brother	Yes	13.1	46.3	72.0	20.4	28.3	6.43	No	ND	-α^-3.7^/-α^-3.7^
2nd brother	No	15.8	48.0	80.0	26.3	32.0	6.00	No	ND	-α^-3.7^/αα

^a^ ND=Not detected

## Discussion

Because there are two genes for β-globin, an individual heterozygous for a β globin variant would be expected to have equal proportions of normal and abnormal hemoglobins. However, some β variants are synthesized significantly less than normal β globin, so the level of these variants (such as E, Lepore, Knossos, K-Woolwich and Vicksburg) would be below that of normal Hb A-a situation associated with the thalassemic phenotype. In heterozygous individuals with forms of unstable hemoglobins such as Koln and Zurich, the proportion of Hb variants is also below that of normal hemoglobin. Differences between the levels of normal and other variant hemoglobins reflect the different tendencies of variants assemble with normal alpha subunits.[1]

In heterozygous forms of hemoglobinopathies with the β variant chain, the normal β chain has a greater affinity to combine with the α chain and form Hb A1 compared with the abnormal β chain, so the amount of Hb A1 is usually greater than abnormal hemoglobin. This situation is more obvious in individuals with both α-thalassemia and a heterozygous form of hemoglobinopathy, because the normal β chain has a stronger tendency to assemble with the deficient α chain present in α-thalassemia and is thus more successful in forming Hb A1 than in individuals with only heterozygous forms of hemoglobinopathies.

The J-Iran variant (β77 His→Asp), first reported in Iran,[2] was subsequently found in Turkey[3] and in a Russian-Armenian family.[4]The substitution at β77 leads to a higher negative charge on the surface of the βJ-Iran subunit, which enhances its electrostatic attraction for the normal positively charged α subunit. As a result, red cells in heterozygous individuals contain more Hb J-Iran than Hb A. In the presence of α- thalassemia, the more attractive βJ-Iran subunit will be even more successful in combining with the deficient α subunits, so that even more Hb J-Iran is formed (unlike the situation with other β variants such as Hb S and Hb C). The same mechanism is responsible for the greater proportion of Hb J-Baltimore than Hb A. Competition experiments with the βA and βJ-Baltimore subunits in vitro showed that when α subunits are deficient, Hb J formation would be greater than in individuals without α-thalassemia.[1][5]

In 1987, Rahbar et al. described a 14-year-old Persian girl who had 65% Hb J-Iran, but who also had Hb H disease with three α gene deletions, which probably explains the difference in the amount of Hb J-Iran between this girl and the family reported here. Her two siblings had 50% Hb J-Iran and probably silent or minor α-thalassemia.[1] In Turkey, the first person with Hb J-Iran (β77 His → Asp) was reported in 1986 by Arcasoy et al.[3] Since then, five cases have been reported in the Turkish population, from Ankara, Antalya and Mugla.[6]

The proband, his mother and siblings did not need blood transfusions and were advised to take 5 mg folic acid daily. They had no apparent health problems related to the hemoglobin variant, and we assume that Hb J-Iran functions correctly in their bodies. To investigate Hb J-Iran functioning, O2 saturation and erythropoietin (EPO) levels are useful because when O2 saturation is normal, EPO secretion and thus serum EPO levels are normal. However, when O2 saturation is below normal (as in some hemoglobin disorders), the increased EPO secretion stimulates red blood cell production to compensate for this defect. When the compensatory mechanism operates, no anemia or apparent health problems are apparent aside from an increase in serum EPO levels.
